# Loss-of-function uORF mutations in human malignancies

**DOI:** 10.1038/s41598-018-19201-8

**Published:** 2018-02-05

**Authors:** Julia Schulz, Nancy Mah, Martin Neuenschwander, Tabea Kischka, Richard Ratei, Peter M. Schlag, Esmeralda Castaños-Vélez, Iduna Fichtner, Per-Ulf Tunn, Carsten Denkert, Oliver Klaas, Wolfgang E. Berdel, Jens P. von Kries, Wojciech Makalowski, Miguel A. Andrade-Navarro, Achim Leutz, Klaus Wethmar

**Affiliations:** 1 Max-Delbrueck-Center for Molecular Medicine, Robert-Roessle-Str. 10, 13125 Berlin, Germany; 2 Charité University Medicine Berlin, Campus Virchow-Klinikum, Berlin-Brandenburg Center for Regenerative Therapies, Augustenburger Platz 1, 13353 Berlin, Germany; 3 Leibniz Institute fuer Molekulare Pharmakologie, Robert-Roessle-Str. 10, 13125 Berlin, Germany; 4 Institute of Bioinformatics, University of Muenster, Niels-Stensen-Straße 14, 48149 Muenster, Germany; 5 Carl-Thiem-Klinikum, 2. Medizinische Klinik, Thiemstr. 111, 03048 Cottbus, Germany; 6 Charité Comprehensive Cancer Center, Charitéplatz 1, 10117 Berlin, Germany; 7 Helios Klinikum Berlin-Buch, Schwanebecker Chaussee 50, 13125 Berlin, Germany; 8 Charité University Medicine Berlin, Institute of Pathology, Chariteplatz 1, 10117 Berlin, Germany; 9 University Hospital Muenster, Department of Medicine A, Hematology, Oncology and Pneumology, Albert-Schweitzer-Campus 1, 48149 Muenster, Germany; 10 Johannes-Gutenberg University of Mainz, Institute of Molecular Biology, Ackermannweg 4, 55128 Mainz, Germany; 11 Humboldt-University, Department of Biology, Invalidenstr. 43, 10115 Berlin, Germany

## Abstract

Ribosome profiling revealed widespread translational activity at upstream open reading frames (uORFs) and validated uORF-mediated translational control as a commonly repressive mechanism of gene expression. Translational activation of proto-oncogenes through loss-of-uORF mutations has been demonstrated, yet a systematic search for cancer-associated genetic alterations in uORFs is lacking. Here, we applied a PCR-based, multiplex identifier-tagged deep sequencing approach to screen 404 uORF translation initiation sites of 83 human tyrosine kinases and 49 other proto-oncogenes in 308 human malignancies. We identified loss-of-function uORF mutations in *EPHB1* in two samples derived from breast and colon cancer, and in *MAP2K6* in a sample of colon adenocarcinoma. Both mutations were associated with enhanced translation, suggesting that loss-of-uORF-mediated translational induction of the downstream main protein coding sequence may have contributed to carcinogenesis. Computational analysis of whole exome sequencing datasets of 464 colon adenocarcinomas subsequently revealed another 53 non-recurrent somatic mutations functionally deleting 22 uORF initiation and 31 uORF termination codons, respectively. These data provide evidence for somatic mutations affecting uORF initiation and termination codons in human cancer. The insufficient coverage of uORF regions in current whole exome sequencing datasets demands for future genome-wide analyses to ultimately define the contribution of uORF-mediated translational deregulation in oncogenesis.

## Introduction

Ribosome profiling and numerous observations on individual transcripts characterized upstream open reading frames (uORFs) as repressive *cis*-regulatory elements, constitutively reducing translation rates of downstream main protein coding sequences (CDSs)^1–4^. A uORF is defined by a translational initiation codon preceding the CDS and a subsequent in-frame termination codon (uStop). Approximately 55% of human transcript leader sequences (TLSs) contain one or more AUG-initiated uORFs, which may precede or overlap the CDS initiation site^5^. During canonical cap-dependent translation, ribosomes frequently initiate at upstream AUG (uAUG) codons, resulting in reduced translation of the associated downstream CDS^3,6^. Accordingly, uORFs regulate translation rates at the CDS by various mechanisms, including consumption of ribosomal pre-initiation complexes, induction of ribosome stalling at uStop codons, and nonsense-mediated mRNA decay^7^.

The regulatory impact of individual uORFs is highly dependent on transcript-specific features, including the position of the uORF within the TLS, its length and the quality of the uAUG-surrounding Kozak consensus sequence (uKozak). The uKozak sequence is optimal for ribosomal initiation in the context of GCCGCC**AUG**R (with core Kozak bases underlined and R representing a purine base)^8^. Protein synthesis from uORF-bearing transcripts requires ribosomes to bypass the uORF initiation codon by leaky scanning or to re-initiate translation after termination at a uStop codon. Despite a mostly repressive effect on downstream translation, specific uORFs mediate a variety of molecular responses, including the translational induction of key regulatory proteins during the integrated stress response^9,10^, the adjustment of protein levels in response to uORF-specific co-regulators^11^, and the control of balanced protein isoform expression from a single transcript^12^.

Defective uORF-mediated translational control alters physiological processes across species from yeast^9^ to mice^13^. Furthermore, several human diseases result from the translational induction of proteins through a mutational loss of uORF initiation codons, as exemplified by Marie Unna hereditary hypotrichosis^14^ and hereditary thrombocytosis^15^. To date, two mutations that enhance uORF-mediated repression in tumor suppressor genes have been linked to the development of human cancer. A point mutation found in hereditary melanoma introduces a uORF in the *CDKN2A* gene resulting in decreased translation of the encoded CDK4/CDK6 kinase inhibitor^16^. Similarly, a 4-bp deletion within a uORF in the tumor suppressor gene *CDKN1B* caused lengthening of the uORF and enhanced repression of the cyclin-dependent kinase inhibitor p27^KIP1^ in a patient with multiple endocrine neoplasia syndrome type IV^17^. Despite such indications for a potential role of uORF mutations in tumorigenesis, no comprehensive search for cancer-related uORF mutations has been performed.

We recently observed constitutive uORF-mediated translational repression of a number of human tyrosine kinases and other proto-oncoproteins, hinting at a mechanism of enhanced proto-oncogene expression through loss-of-function uORF mutations in cancer development^5^. Here, we developed a screening approach combining PCR amplification and deep sequencing to simultaneously investigate selected uORF start site regions in various cancer samples of different entities. We screened 308 human malignancies for loss-of-uAUG mutations in 404 uORF initiation sites of 132 potential proto-oncogenes. Additionally, we analyzed 464 whole exome sequencing datasets of colon cancer for mutations in uORF-related initiation and termination codons. Our data revealed a number of non-recurrent loss-of-uAUG, loss-of-uStop and uKozak-affecting mutations in various types of cancer and suggest that genetic defects in uORF-mediated translational regulation may contribute to malignant transformation *in vivo*.

## Results and Discussion

### A PCR-based, multiplex identifier-tagged deep sequencing approach for the identification of uORF mutations

**Figure 1:**
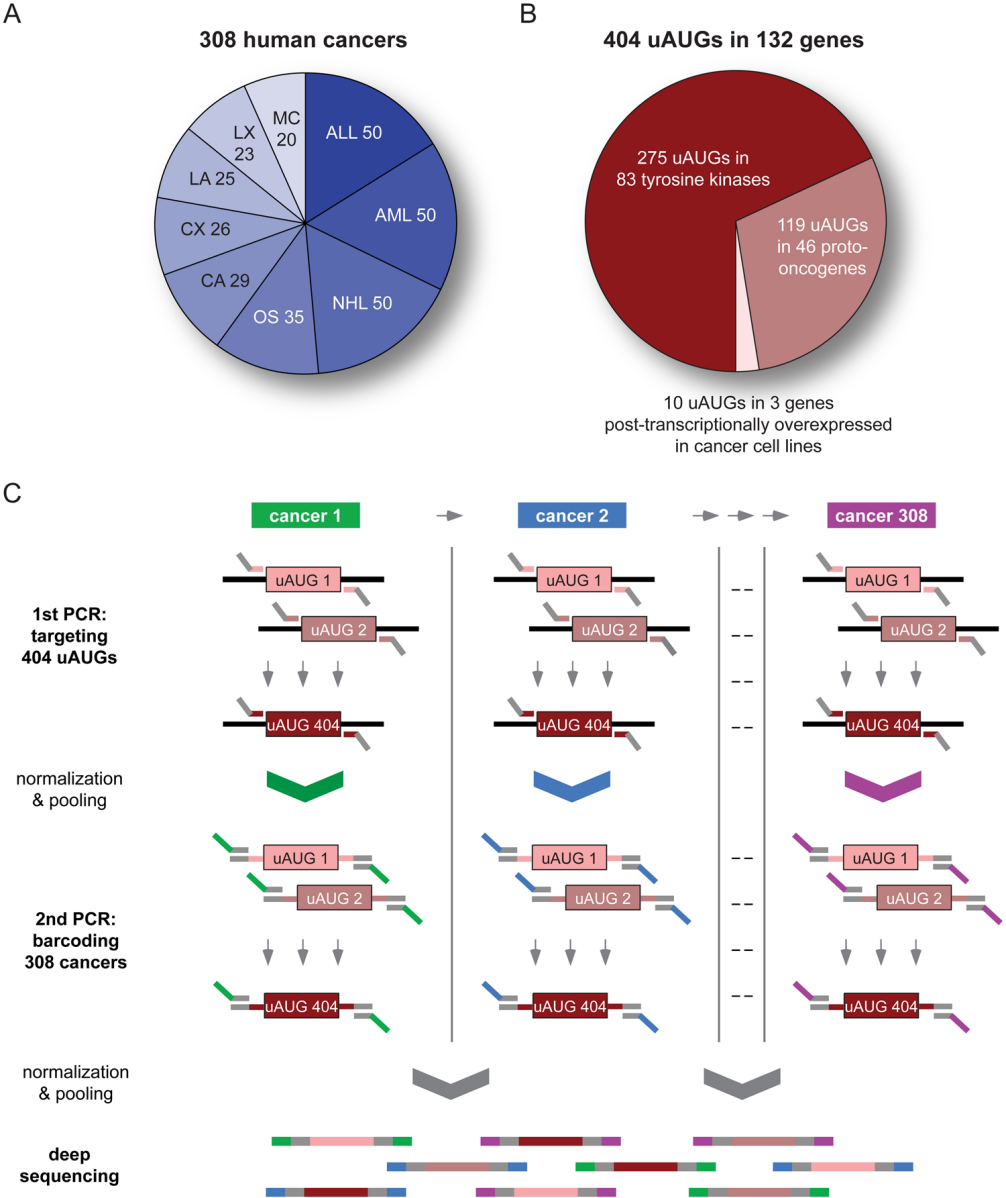
Target sets and workflow of the PCR-based, multiplex identifier-tagged deep sequencing approach. (**A**) Composition of the cancer sample set with numbers indicating sample sizes of investigated malignant entities: ALL – acute lymphoblastic leukemia, AML – acute myeloid leukemia, NHL – non-Hodgkin lymphoma, OS – osteosarcoma, CA – colon adenocarcinoma, CX – colon xenograft, LA – lung adenocarcinoma, LX – lung xenograft, MC – mammary carcinoma. (**B**) Composition of the target gene set consisting of indicated numbers of uORF-bearing tyrosine kinases^5^, previously validated proto-oncogenes^18^ and genes post-transcriptionally induced in cancer cell lines^19^ (see also Supplementary Table 1). (**C**) Flow chart displaying amplification and normalization steps allowing simultaneous deep sequencing of 404 uORF initiation sites of 132 target genes in 308 individual cancer samples. Briefly, genomic regions of uAUG targets were amplified individually from every cancer DNA (see also Supplementary Table 7). uAUG-specific amplicons of each cancer sample were pooled and labeled with cancer-specific MID-tags in a second round of PCR (see also Supplementary Table 8). After normalization and pooling of all MID-tagged amplicons, a deep sequencing library was generated and analyzed using the Illumina® HiSeq2000 sequencing system.

To systematically search for genetic alterations affecting uORF initiation codons (uAUGs) and related Kozak consensus sequences (uKozaks), a PCR-based, multiplex identifier (MID)-tagged deep sequencing approach was established (Fig. 1). We included 308 hematologic and solid tissue malignancies, comprising samples derived from acute myeloid leukemia (AML, 50 cases), acute lymphoblastic leukemia (ALL, 50), non-Hodgkin lymphoma (NHL, 50), osteosarcoma (OS, 35), mammary carcinoma (MC, 20), lung (LA, 25) and colon (CA, 29) adenocarcinoma, and a number of mouse xenografts of human colon tumors (CX, 26) and lung adenocarcinoma (LX, 23) (Fig. 1A). Pre-test histological or cytological analysis confirmed a tumor cell content ≥50% for each sample. To define the uORF-target set, we mapped genomic positions of all uORF initiation codons in 132 cancer-associated genes (Fig. 1B, Supplementary Table 1), comprising 83 human tyrosine kinases (TKs)^5^, 46 validated proto-oncogenes overexpressed or amplified in human cancer^18^, and three candidate genes post-transcriptionally induced in cancer cell lines^19^. Target regions were amplified from cancer-derived DNA in a first round of PCR using uAUG-specific oligonucleotides containing a 5′-universal linker sequence (Fig. 1C). After quantitative normalization, all uAUG-specific amplicons of the same cancer sample were pooled and subjected to a second round of PCR adding a MID sequence tag via the previously attached universal linker sequence. Finally, MID-tagged cancer-specific pools were again normalized for DNA content, combined, and used to generate a single multiplex library for deep sequencing. As a whole, the screening protocol enabled the simultaneous investigation of 404 uORF translational start sites in 308 human cancer samples, equaling 124,432 individual genomic regions.

### Identification of uORF-associated genetic alterations in human malignancies

**Figure 2:**
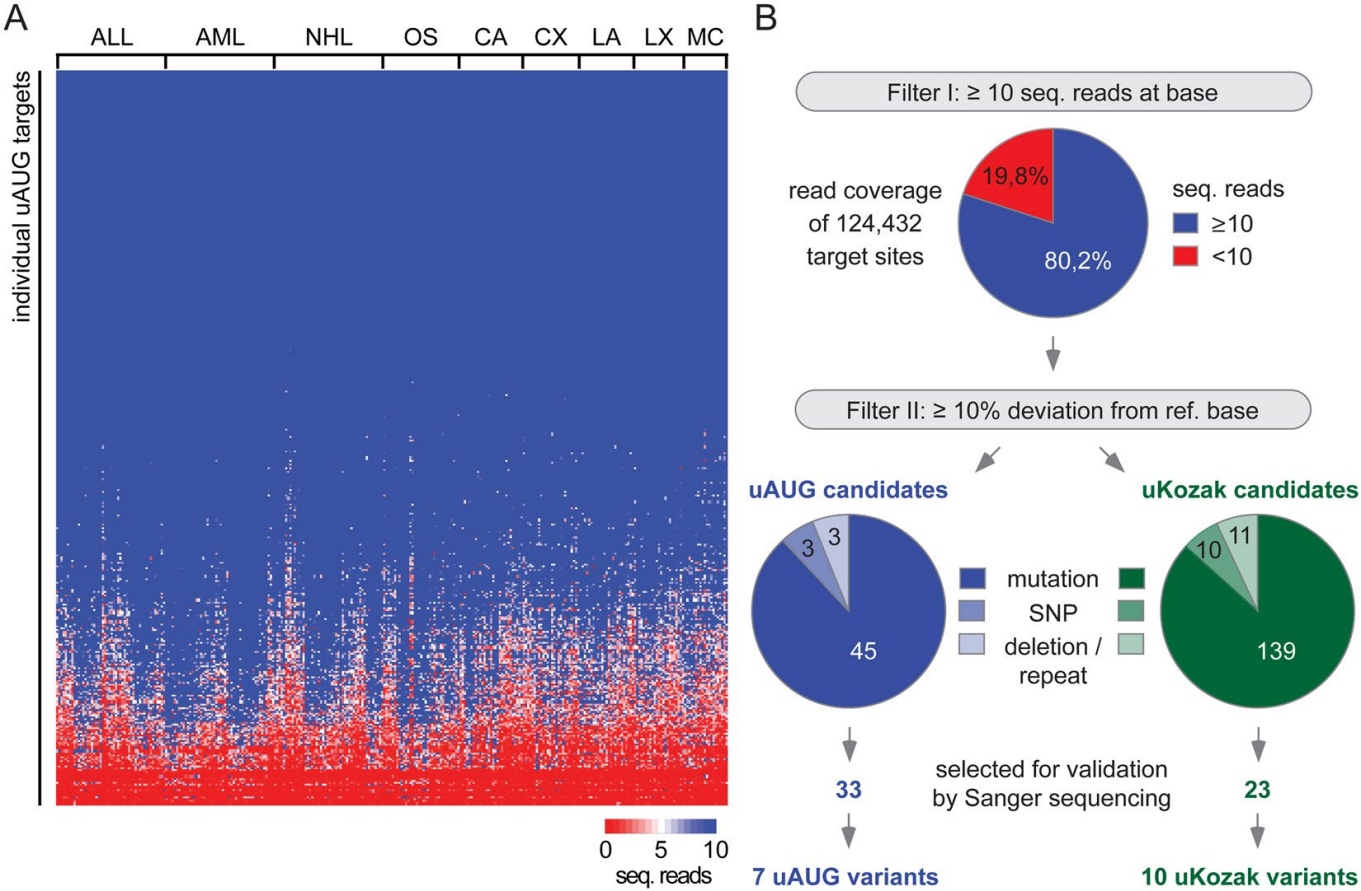
Recovery of genetic information of targeted uAUG regions and identification of uORF-associated alterations in human cancers. (**A**) Heatmap displaying the number of sequencing reads for individual uAUG target sites (rows) and individual cancer samples of indicated entities (columns). The threshold was set to ≥10 sequencing reads (seq. reads) (see also Supplementary Table 2). (**B**) Summary of sequencing data processing. The top pie chart shows the proportion of all individual target sites (404 uAUGs of 308 cancer samples) that were covered by ≥10 sequencing reads. The bottom pie charts represent the numbers of potential genetic alterations (mutations, single nucleotide polymorphisms (SNPs) and long deletion/repeat regions) in uAUG and uKozak target sites that showed ≥10% deviation from the reference base (ref. base)(see also Supplementary Tables 3 and 4). Selected candidate mutations were subsequently re-sequenced by Sanger confirming the indicated number of uORF-associated alterations.

Approximately 120 million sequencing reads with sample-specific MID-tags were generated and could be assigned unequivocally to individual cancers. All reads were computationally aligned to the human reference genome (hg19), resulting in recovery of all, except two, of the expected uAUG-specific amplicons. The median target-specific read coverage of 103 was lower as compared to the expected average of approximately 1000 reads per target region and showed high variation in site-specific read counts (0 to 160,060 reads per site). Despite thorough quantitative adjustments after the first and second round of amplification, 45% of total sequencing reads mapped to the top 5% of covered target sites (Supplementary Table 2). Across cancer samples the distribution of reads per amplicon was similar (Fig. 2A, Supplementary Table 2), suggesting that site-specific efficiencies of individual PCRs accounted for the variance in read coverage, rather than differences in DNA quality. After all 80.2% of the 124,432 individual genomic regions were covered by 10 or more sequencing reads (Fig. 2A,B, top) and qualified for further analysis.

Nucleotide positions with more than 10% deviation from the reference genome were classified as potential uORF variant calls, as a heterozygous mutation in a sample containing 50% malignant cells would result in a 25% deviation of reads and cancer samples may be genetically diverse. Applying these filters, sequence alignments identified 45 candidate mutations affecting uAUGs and 139 candidate mutations affecting uKozak sequences (Fig. 2B, bottom; Supplementary Tables 3 and 4). Additionally, 6 and 21 annotated uORF-associated single nucleotide polymorphisms (SNPs) as well as long deletion/repeat regions^20^ were detected in uAUG codons and uKozak sequences, respectively. Selected Sanger re-sequencing of independently generated uAUG-specific amplicons validated the presence of three out of three uAUG-deleting SNPs and eight out of nine uKozak SNPs (Table 1, Supplementary Tables 3 and 4), demonstrating the suitability of our approach to identify genetic variants at single nucleotide resolution.

**Table 1:** Summary of verified uORF-associated SNPs.

**Gene**	**Chromosome coordinates**	**Reference base**	**SNP base**	**SNP freq [%]**	**uAUG/uKozak variation**	**Number of affected cancer samples**	**Related entities**
CAMKK2	chr12:121735975	A	—	1.4	**A**TG	2	CA
EPHA3	chr3:89156859	A	T	0.2	**T**TG	1	ALL
KDR	chr4:55991731	A	G	55.4	**G**TG	4	MC, ALL, AML, OS
CAMKK2	chr12:121712374	G	A	n.a.	tgcC**a**gATGG	1	AML
MDM2	chr12:69202164	A	G	33.7	agtGgaATG**G**	237	all entities
MUSK	chr9:113431103	T	C	1.2	caaAg**c**ATGC	2	AML, NHL
NRP2	chr2:206547747	T	C	0.6	acaTa**c**ATGC	5	MC, CA, AML, NHL
PTK2B	chr8:27179964	C	T	0.05	**t**ggCagATGA	1	AML
STAT6	chr12:57505073-8	GTGTGT	—	div.	**gtgTgt**ATGTATGT	306/307/265/273/138/146	all entities
TTN	chr2:179672033	G	A	2.6	tc**a**GagATGG	2	CA, LX
TYK2	chr19:10490402	T	C	16.5	c**c**tTtgATGG	120	all entities
YEATS4	chr12:69753557	G	A	0.2	gccT**a**aATGG	1	OS

The table shows confirmed annotated SNPs (in bold) in uAUGs (top) and uKozak sequences (bottom, uAUG is underlined and core uKozak bases are in capital letters) with information of affected genes and cancer samples.

Note that the single nucleotide deletion in the uAUG of *CAMKK2* and the 6-bp repeat deletion in the uKozak sequence of *STAT6* did not alter the uORF start site or uKozak sequence, respectively, as resulting genotypes correspond to the reference base(s). In the case of STAT6, different numbers of affected cancer samples were determined for each base in the 6-bp repeat region.

freq – frequency; n.a. – not annotated; div. – diverse annotations; all entities – ALL, AML, NHL, OS, CA, CX, LA, LX, MC.

We then selected 33 uAUG and 23 uKozak candidate mutant sites for validation by Sanger re-sequencing after manual exclusion of cases, where low read numbers and variable nucleotide substitutions within the same sample indicated probable false positive mutation calls (Fig. 2B, Supplementary Tables 3 and 4). Finally, Sanger re-sequencing confirmed five novel uORF-associated mutations in individual cancer samples, resulting in the loss of a uORF initiation codon in four cases and in the alteration of the uKozak sequence in one individual (Table 2, Supplementary Tables 3 and 4). The distribution of sequencing reads indicated heterozygous loss-of-uAUG mutations in various cancers, affecting the Src family tyrosine kinase BLK proto-oncogene (*BLK*) in a colon adenocarcinoma, the ephrin receptor B1 (*EPHB1*) in a mammary carcinoma and a colon cancer xenograft, the Janus kinase 2 (*JAK2*) in a sample of chronic lymphocytic leukemia, and the mitogen-activated protein kinase kinase 6 (*MAP2K6*) in a colon adenocarcinoma. Additionally, the uKozak mutation affected the chromodomain helicase DNA binding protein 1-like gene (*CHD1L*) in a colon cancer xenograft. The somatic origin of uORF-related mutations was confirmed for the *BLK* and the *CHD1L* mutants, while the mutation observed in *MAP2K6* was found to be a germline variant by analyzing normal tissue controls of affected patients (Supplementary Table 5). The mutations observed in *EPHB1* and *JAK2* could not be further characterized due to the lack of normal tissue.

**Table 2:** Summary of verified uORF-associated novel mutations.

**Gene**	**Chromosome coordinates**	**Reference base**	**uAUG/uKozak mutation**	**Number of affected cancer samples**	**Related entities**
BLK	chr8:11351560	G	AT**T**	1	CA
EPHB1	chr3:134514263	A	**G**TG	2	MC, CX
JAK2	chr9:5021969	G	AT**A**	1	NHL
MAP2K6	chr17:67410881	T	A**C**G	1	CA
CHD1L	chr1:146731516	G	ttgTg**t**ATGA	1	CX

The table shows newly identified mutations (in bold) in uAUGs (top) and uKozak sequences (bottom, uAUG is underlined and core uKozak bases are in capital letters) with information of the affected genes and cancer samples.

### Loss-of-uAUG mutations in EPHB1 and MAP2K6 induce downstream translation

**Figure 3:**
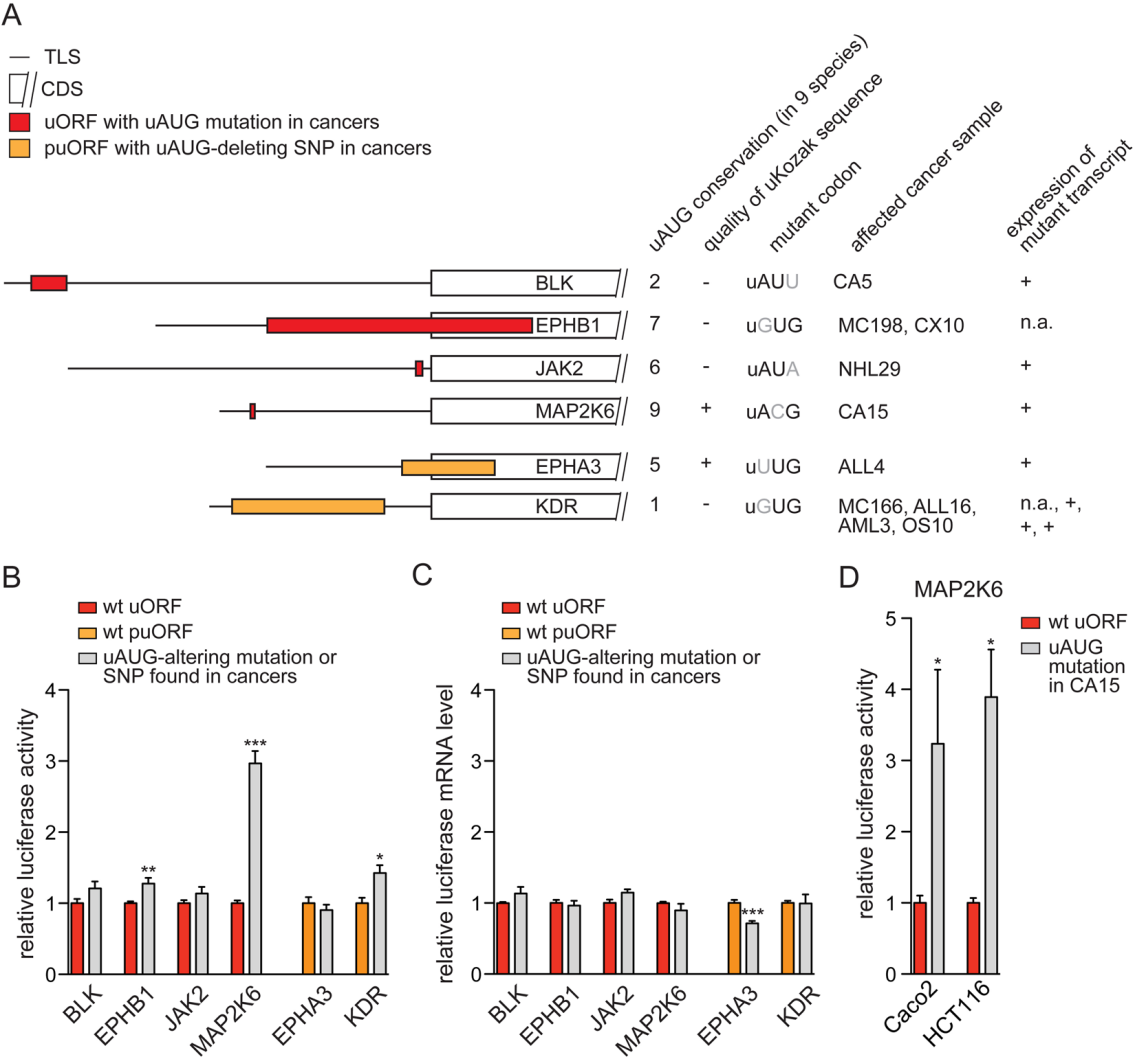
Translational impact of identified loss-of-uORF mutations. (**A**) Schematic representation showing the position and length of uORFs with identified uAUG-associated mutations and polymorphisms (p) in the indicated transcripts. Conservation of affected uORF start sites among nine vertebrate species (human, rhesus, mouse, rat, cow, dog, elephant, chicken, and zebrafish) is indicated and the quality of uKozak contexts is depicted as intermediate (+, one core uKozak base match) or weak (−, no core uKozak base match). Additional columns display the detected sequence of the mutant codon, the affected cancer sample and the expression of indicated transcripts in affected cancer samples determined by semi-quantitative real-time PCR (see also Supplementary Fig. 1). Note that all transcripts contained additional uORFs that were devoid of genetic alterations and are not illustrated here (see also Supplementary Table 6 and Supplementary Fig. 2). n.a. - not analyzed due to the lack of cancer material. (**B**,**C**) Luciferase assays and real-time PCR analysis in HeLa cells showing relative luciferase activities and mRNA levels in the presence of indicated TLSs containing wild-type (red or orange) or mutant (gray) uORF initiation sites as shown in (**A**). (**D**) Luciferase assays demonstrating relative luciferase activities in the presence of the wild-type (red) or mutant (gray) uORF initiation codon in the TLS of *MAP2K6* in two indicated colon cancer-derived cell lines. Error bars represent means ± standard error of the mean (s.e.m) of Firefly luciferase signals relative to Renilla luciferase internal control signals from duplicate measurements of at least three (b,c) and two (d) independent experiments. Statistical significance was determined by the two-tailed, non-parametric Mann-Whitney test and is indicated by *P < 0.05, **P < 0.01 and ***P < 0.001. Numbers identify the specific cancer sample affected by the uAUG alteration.

Focusing on genetic variants that functionally ablated uORF initiation codons, we analyzed the expression of related transcripts in the original cancers. Wherever primary material was available, the affected transcripts were readily detected by semi-quantitative real-time PCR, suggesting that the observed uORF-altering mutations may have been translationally active *in vivo* (Fig. 3A and Supplementary Figure 1). To monitor the translational impact of the loss-of-function uORF alterations, TLSs containing either the wild-type (wt) or the disrupted uORF initiation site were introduced into a luciferase uORF-reporter system as previously described^5^ (Fig. 3A, Supplementary Table 6). Two mutations, comprising the uAUG to uGUG mutation in *EPHB1* and the uAUG to uACG mutation in *MAP2K6*, as well as the uAUG-deleting SNP variant in *KDR*, induced enhanced translation of the downstream coding sequence to various extends (Fig. 3B), while the uAUG-ablating alterations in *BLK*, *JAK2* and the SNP in *EPHA3* showed no detectable effects on reporter expression. The loss-of-uAUG mutation in *EPHB1* was associated with a mild enhancement of reporter expression (approximately 30%), whereas the uORF-disrupting mutation in *MAP2K6* caused a marked induction of translation (approximately 3-fold). This was within the range of translational induction observed for other uORF-regulated transcripts^5,6^. For both, *EPHB1* and *MAP2K6*, wt and mutant transcript levels did not differ significantly, excluding the possibility that changes in transcription or mRNA stability accounted for the observed variation of protein levels (Fig. 3C).

Thus, our targeted screening approach revealed unprecedented experimental evidence for rare loss-of-uAUG mutations in human cancer. In analogy to non-malignant diseases^14,15^, the genetic ablation of functionally active uORFs may contribute to malignant transformation in individual cases by inducing overexpression of a related downstream oncoprotein. Depending on the type of cancer, EPHB1 has been linked to tumor-suppressor and oncogenic functions by affecting major cellular programs including cell cycle control, apoptosis, regulation of cell-cell contacts, and migration^21–23^. For *MAP2K6*, two previous reports suggested a proto-oncogenic role of MAP2K6 overexpression in various types of solid cancer, including colon adenocarcinoma^24,25^, yet, the mechanism of the tumor-specific elevation of MAP2K6 protein levels was not investigated. As the *MAP2K6* mutation identified in our screen originated from a colon adenocarcinoma sample, we performed additional luciferase assays in the colon cancer-derived cell lines Caco2 and HCT116. Similar to the results observed in HeLa cells, the loss-of-function uORF mutation proved to be translationally active in colon cancer cells (Fig. 3D). Of note, the *MAP2K6* uORF mutation was observed in the normal control tissue of the affected patient as well, suggesting a germline transmission of the variant allele that may have encompassed predisposition to tumor development. However, no further information on the patient’s family history was available. Very recently, the *MAP2K6* uAUG variant has been observed in an independent whole genome sequencing analysis (SNP rs751306749)^20^, yet the functional role and frequency of this variant remains obscure.

As shown before, the uAUG-deleting SNP variant in *KDR* resulted in a mild de-repression of downstream translation^5,6^. In a previous report, elevated KDR protein levels were observed in lung cancerous tissue in association with the loss-of-function uORF allele, indicating functionality of the mutant transcript in a tumor environment^26^. Moreover, the uORF-disrupting SNP in *KDR* has been associated with a trend toward shorter overall survival of pancreatic carcinoma patients^27^; however, the high frequency of the *KDR* loss-of-uORF allele observed in the 1000 genomes analysis does not support a driving role of this SNP (rs7667298) during tumor formation^28^.

Given the high variability of uORF regulatory functions described in the literature^7^, the detection of non-functional uORF mutants (*BLK*, *JAK2* and *EPHA3)* was expected. The lack of translational activity may be explained by transcript-specific features, including the degree of evolutionary conservation of the uAUG, the quality of the uKozak vicinity, or the presence of additional uORFs within the same mRNA (Supplementary Fig. 2). Moreover, single nucleotide exchanges in an uAUG always result in a near-cognate non-uAUG codon that may occasionally serve as alternative uORF-initiating codon to sustain the inhibitory effect of the original uORF on downstream translation^29,30^. Apart from transcript immanent features, global translational conditions and the abundance of translational co-factors may differentially affect individual uORF start sites. In the context of carcinogenesis, recent observations linked uORF-mediated activation of translation to the abundance of eukaryotic initiation factors (eIFs) induced by various oncogenic signaling pathways. Both, eIF2A and eIF6 were shown to specifically direct translation towards complex GC-rich and uORF-bearing mRNAs^31^. Especially, eIF6 is overexpressed in several human cancers including colon cancer^32^ and contributed to malignant transformation and tumor growth *in vivo*^33,34^. Thus, beyond a direct induction of downstream translation through loss-of-uAUG mutations, uORFs may serve as sensors of oncogenic signaling, similar to their global role in directing nonsense-mediated decay towards uORF-bearing transcripts during stress responses^35^.

After all, the non-recurrent nature of the uORF-associated genetic alterations detected by our targeted sequencing approach precluded extensive experimental investigations on the transforming capacity of individual mutations and emphasized the need to increase the number of samples for individual cancer types.

### Whole exome sequencing uncovers additional non-recurrent loss-of-uAUG mutations in colon cancer

We extended our study to 464 colon adenocarcinoma-derived whole exome sequencing datasets available through NCI´s Genomic Data Commons portal^36^ (https://gdc-portal.nci.nih.gov) to further define the frequency of uORF mutations. The genomic positions of the previously identified uORF-mutations in BLK, EPHB1, JAK2, and MAP2K6 were covered by ten or more reads in 6, 44, 438, and 141 cancer samples, respectively. None of four independent SNP calling tools (SomaticSniper, VarScan2, MuTect2, and MuSE) detected additional cases of these variants in the extended colon cancer sample set (data not shown). Given the high coverage for the JAK2 and MAP2K6 uORF initiation sites, we conclude that the JAK2 and MAP2K6 uORF mutations appeared to be non-recurrent in colon cancer.

We also analyzed the exome sequences of tumor samples and corresponding normal controls for somatic mutations in all 59818 uORF initiation codons and 47108 associated uORF termination codons, as identified in the latest human genome assembly (hg38). The number of uStop codons was lower as compared to the uAUGs, because subsequent in-frame uAUGs may share the same termination codon. Furthermore, the analysis did not include uORF-related Stop codons located downstream of the respective CDS start site. On average, approximately 41% of the upstream initiation and termination codons were covered by ten or more reads in both, tumor samples and normal controls (Supplementary Figure 3). These data demonstrate that uORF regions are still insufficiently covered in current whole exome sequencing datasets, as classical exome sequencing mostly focused on protein coding regions and thus neglected substantial parts of the TLSs.

Nevertheless, the four SNP calling tools reported variable numbers of somatic variants, ranging from 100 for the MuSE tool to 1940 for MuTect2 tool (Supplementary Figure 3). To maximize specificity, we filtered for SNPs that had been identified by all four platforms resulting in the detection of 61 non-recurrent somatic mutations (Supplementary Table 9). None of these showed genetic variance among the 1000 genomes dataset. Eight variants had been annotated for genetic variability in dbSNP (NCBI) before^20^. The remaining 53 novel mutations represented 22 single nucleotide variants (SNVs) functionally deleting a uAUG and 31 SNVs functionally deleting a uStop codon, respectively. Again, the non-recurrent nature of these variant alleles precluded the in-depth functional analysis of individual cases.

### Conclusions and future prospects

In summary, our systematic PCR-based re-sequencing approach of uORF start sites in more than 300 samples derived from seven distinct cancer entities provides initial experimental evidence for rare loss-of-function uORF mutations in human malignancies. Targeting <1% of currently annotated uORF initiation codons, we may have missed recurrent uORF variants, as the choice of uORFs was biased by previous classifications suggesting a potential uORF-related proto-oncogenic role of the selected genes. The absence of recurrent somatic mutations in both, the compilation of distinct cancer entities in the PCR-based screen and in the larger cohort of colon adenocarcinomas analyzed by whole exome sequencing, may argue against a pivotal role of deregulated uORF-mediated translational control in carcinogenesis. Similarly, we and others did not identify recurrent mutations in uORF initiation codons in cancer by exploring publicly available databases of genetic variations (ClinVar, COSMIC and TCGA)^37^. Notwithstanding, our data add loss-of-function uORF mutations to the list uORF-mediated mechanisms of translational control, including (I) the adaption of protein expression to nutrient supply and growth factor signaling, (II) the activation of mRNA decay through uStop-induced ribosome stalling, (III) the regulation of protein isoform expression through uORF-directed alternative start site selection, and (IV) the direct interaction of co-factors with the nascent uORF-derived peptide (summarized in^7^). The identification of loss-of-function uORF mutations in human malignancies emphasizes the need for comprehensive analyses of uORF regions in the growing number of cancer-derived whole genome sequencing datasets. Such efforts will ultimately allow to define the role of uORF-related genetic variability in tumorigenesis.

## Methods

### Patient material

Fresh frozen cancer tissue samples and selected matched normal tissue controls from patients with a histologically confirmed diagnosis of cancer were obtained from contributors of the Helios Klinikum Berlin-Buch, the Charité Universitaetsmedizin Berlin and the Max-Delbrueck-Center for Molecular Medicine Berlin-Buch and analyzed with approval from with the local ethics committee (Ethikkommision, Charité, Berlin: EA1/003/13). All experiments were performed in accordance with relevant guidelines and regulations. Informed consent was obtained from all participants and/or their legal guardians.

Genomic tumor DNA was extracted using the QIAamp DNA Mini Kit (Qiagen) after histopathological verification of a tumor cell content of at least 50% in samples of acute lymphoblastic leukemia (50), acute myeloid leukemia (50), non-Hodgkin lymphoma (50), colon (29) and lung (25) adenocarcinoma, mammary carcinoma (20), osteosarcoma (35), and colon (26) and lung (23) adenocarcinoma patient-derived xenografts.

### Multiplex identifier-tagged PCR deep sequencing approach

Genetic alterations in uORF initiation sites were analyzed in three target gene sets consisting of (I) previously defined human tyrosine kinases^5^, (II) previously validated proto-oncogenes^18^ and (III) genes identified manually as being post-transcriptionally overexpressed in cancer cell lines from the CellMiner database^19^. We designed and established 367 customized PCR primer pairs (Supplementary Table 7) to amplify 404 uORF initiation sites (including the uKozak context) as mapped by a previous genome-wide sequence analysis^5^ using the Pfu Plus! DNA Polymerase (Roboklon). All primers contained a 5′-extending universal linker sequence (CTCGAGATCT) to facilitate subsequent patient-specific labeling of individual amplicons. uAUG-specific PCRs were prepared using the Tecan Evo Pipetting Workstation equipped with a 384 multichannel pipetting head with disposable tips (Tecan AG, Switzerland).

Based on semi-quantitative gel analysis, similar amounts of uAUG-specific amplicons derived from individual cancer samples were pooled and purified using the Invisorb Spin DNA extraction Kit (Stratec). Patient-specific pools of amplicons were labeled in a second round of PCR, using bipartite primers containing the complementary universal linker sequence and one of 308 previously established multiplex identifier (MID) tag sequences (Supplementary Table 8), allowing robust cancer sample discrimination of individual amplicons^38^.

After another round of agarose-gel-based quantification and spin-column purification, individual MID-tagged pools of uAUG-specific amplicons were combined in similar amounts to generate the final sequencing library by using the TruSeq gDNA Sample Prep Kit. Deep sequencing was performed on an Illumina^®^ HiSeq2000 sequencing system with the TruSeq SBS Kit v3 and the PE (paired-end) Cluster Kit v3, producing read lengths of 2 × 101 nucleotides on average.

Sequencing reads containing cancer-specific MID-tags were aligned to the reference genome (hg19) of the UCSC genome browser database^39^ using bowtie2 v2.0.2^40^. Nucleotide-specific potential base alterations were listed for all uAUG and uKozak bases that were covered by 10 or more patient-specific sequencing reads and showed a minimal deviation from the reference genome in more than 10% of reads. Of the resulting potential uAUG alterations, those with a low probability of representing a true mutation as indicated by variable base substitutions (e. g. seven reads for the reference base A, and one read each for C, G and T) were excluded manually. For all remaining candidate uAUG alterations, uAUG-specific amplicons were regenerated from primary cancer DNA and re-sequenced using the Sanger sequencing services of Eurofins Genomics, Germany.

### Semi-quantitative real-time PCR analysis

Total RNA was extracted from cancer samples (CA5, CA9, CA13, CA15, ALL4, ALL11, ALL16, LA2, LX2, AML3, AML6, NHL29, NHL46, and OS10) using the GeneMATRIX Universal RNA purification Kit (Roboklon). DNAse (Roche)-digested RNA extracts were reverse-transcribed using the RevertAid First Strand cDNA Synthesis Kit (Thermo Scientific). Semi-quantitative detection of distinct mRNA levels and HPRT as an internal control was performed with the Power SYBR Green PCR Master Mix (Applied Biosystems) and customized real-time PCR primers (BLK-RT for: CACTCCCAAGGCTGATTGAC, BLK-RT rev: GCCTCAGACACCAGGATGTT; EPHA3-RT for: AGACAGTTTGCTGCGGTCAG; EPHA3-RT rev: GGATGTTCAGGTTCTTGCCA; HPRT-RT for: AGTCTGGCTTATATCCAACACTTCG, HPRT-RT rev: GACTTTGCTTTCCTTGGTCAGG; JAK2-RT for: GGGTTAACCAAAGTCTTGCCA, JAK2-RT rev: GAGGCCACAGAAAACTTGCTC; KDR-RT for: AGTTGGTGGAACATTTGGGA, KDR-RT rev: TCCAGAATCCTCTTCCATGC; MAP2K6-RT for: GCCTATAATGGAACTGGGACG, MAP2K6-RT rev: GGCTATTTACTGTGGCTCGGA).

### Cell lines

Caco2 (ATCC), HCT116 (ATCC), HeLa (DSMZ) and HEK293 (DSMZ) cell lines were incubated at 37 °C in a humidified 5% CO_2_ incubator. Cells were kept in DMEM (Life Technologies) supplemented with 10% fetal bovine serum (PAA), 1% HEPES (PAA) and 5% penicillin/streptomycin (PAA).

### Generation of constructs for luciferase reporter assays

TLSs containing wild-type (wt) or mutant uORF start sites were synthesized (GeneArt, ThermoFisher Scientific) or generated by PCR amplification and subsequent mutagenesis (Supplementary Table 6). TLS inserts also contained the endogenous transcript-specific CDS start sites, including the core Kozak base + 4. PCR amplification of TLSs was performed using the Pfu Plus! DNA Polymerase (Roboklon) on genomic DNA derived from the HEK293 cell line for *EPHA3*, *EPHB1* and *MAP2K6* and on HEK-derived cDNA for *BLK* together with customized PCR primers harboring overhangs for enzymatic restriction (in capital letters): BLK for: ACGGCTAGCcacacagatggcacatggca, BLK rev: GTGCCGCGGCCATccttggcaatgcttca; EPHA3 for: CACGCTAGCcccgctctgcttcagcgcac, EPHA3 rev: GGACCGCGGCCATgttgctggtgcagagg; EPHB1 for: TGCCCCGGGgtcagtctggccggctccgt, EPHB1 rev: CCCAGATCTCCATcgccggccgacggccc; MAP2K6 for: TTTGCTAGCagttccaagtttggagcttt, MAP2K6 rev: GTTCCGCGGACATtttcccctttcctttg. PCR-amplified or synthesized TLSs excised from purchased vectors were purified using the Invisorb Spin DNA extraction Kit (Stratec) and cloned via flanking restriction sites (*Bgl*II–AGATCT, *Nhe*I–GCTAGC, *Sac*II–CCGCGG, *Sma*I–CCCGGG) into a previously generated, custom-made Firefly luciferase reporter system^5^ using T4 DNA ligase (New England Biolabs). Site directed mutagenesis of uORFs was performed using the Pfu Plus! DNA Polymerase (Roboklon) and customized PCR primers (BLK-mut for: gtggcgttccgctccTTGactgtcgcgcggccg, BLK-mut rev: cggccgcgcgacagtCAAggagcggaacgccac; EPHA3-mut for: tcagtggcatgcttcTTGgagatatgctcctct, EPHA3-mut rev: agaggagcatatctcCAAgaagcatgccactga; EPHB1-mut for: aacacacacacacacGTGcacacccacacccac, EPHB1-mut rev: gtgggtgtgggtgtgCACgtgtgtgtgtgtgtt; MAP2K6-mut for: cagccctggcccatcACGtagctgcagcacagc, MAP2K6-mut rev: gctgtgctgcagctaCGTgatgggccagggctg).

### Luciferase reporter assays

Firefly (custom-made Firefly luciferase vectors with inserted TLSs) and Renilla (pRL-CMV vector, Promega) luciferase activities and mRNA levels were measured in luciferase reporter assays and real-time PCR analysis (Firefly for: ATCCATCTTGCTCCAACACC, Firefly rev: TCGCGGTTGTTACTTGACTG; Renilla for: GGAATTATAATGCTTATCTACGTGC, Renilla rev: CTTGCGAAAAATGAAGACCTTTTAC) as described previously^5^. Briefly, HeLa, HEK, Caco and HCT116 cells were seeded and cultured under standard conditions. After 6 h, cells were transfected with 1 μg/12-well of the TLS-Firefly luciferase reporter construct and 30 ng of Renilla luciferase reporter construct using Metafectene transfection reagent (Biontex). 42 h later, Firefly and Renilla luciferase activities and mRNA levels were determined. For each construct, Firefly luciferase signals were normalized to Renilla luciferase internal control signals.

### Determinating genomic coordinates of uORFs in hg38

A custom Python script was used to retrieve uORF coordinates (available at https://bitbucket.org/TabeaK/uorf-finder). The script uses the RefSeq transcript annotation file downloaded from the UCSC Genome Browser to get the chromosome coordinates of all genes. For our analysis the RefSeq transcripts of the hg38 human genome assembly were used (UCSC table “refGene”, 66656 transcripts on 2016-11-17). The script excludes transcripts that do not have any annotated CDS. It then extracts the TLS sequence, excluding all intronic regions that might intersect the TLS, and searches for uAUG and uStop codons (TAA, TAG, TGA). For each uAUG the script identifies the closest in-frame uStop and reports the uORFs chromosome coordinates in a BED file.

### Whole exome sequencing analysis

On November 3^rd^ 2016, we filtered the gdc sequencing data portal (https://portal.gdc.cancer.gov/) for whole exome sequencing datasets derived from colon adenocarcinoma samples. Variant allele frequencies were available for 492 sample pairs originating from 433 individual cancers and normal tissue controls, respectively. Four different SNP calling tools (SomaticSniper at http://gmt.genome.wustl.edu/packages/somatic-sniper/, VarScan2 at http://dkoboldt.github.io/varscan/index.html, MuTect2 at https://software.broadinstitute.org/gatk/gatkdocs/org_broadinstitute_gatk_tools_walkers_cancer_m2_MuTect2.php, MuSE at http://bioinformatics.mdanderson.org/main/MuSE) were used in the ‘matched mode´, comparing tumor and normal datasets. All vcf files were subsetted to the genomic positions of all uAUG and uStop positions and analyzed for SNPs that were shared between the different SNP callers.

## Electronic supplementary material

Supplementary Information

Supplementary Table 1

Supplementary Table 2

Supplementary Table 3

Supplementary Table 4

Supplementary Table 5

Supplementary Table 6

Supplementary Table 7

Supplementary Table 8

Supplementary Table 9
