# Author Correction: Loss-of-function uORF mutations in human malignancies

**DOI:** 10.1038/s41598-021-82176-6

**Published:** 2021-01-28

**Authors:** Julia Schulz, Nancy Mah, Martin Neuenschwander, Tabea Kischka, Richard Ratei, Peter M. Schlag, Esmeralda Castaños-Vélez, Iduna Fichtner, Per-Ulf Tunn, Carsten Denkert, Oliver Klaas, Wolfgang E. Berdel, Jens P. von Kries, Wojciech Makalowski, Miguel A. Andrade-Navarro, Achim Leutz, Klaus Wethmar

**Affiliations:** 1 Max-Delbrueck-Center for Molecular Medicine, Robert-Roessle-Str. 10, 13125 Berlin, Germany; 2 Charité University Medicine Berlin, Campus Virchow-Klinikum, Berlin-Brandenburg Center for Regenerative Therapies, Augustenburger Platz 1, 13353 Berlin, Germany; 3 Leibniz Institute Fuer Molekulare Pharmakologie, Robert-Roessle-Str. 10, 13125 Berlin, Germany; 4 Institute of Bioinformatics, University of Muenster, Niels-Stensen-Straße 14, 48149 Muenster, Germany; 5 Carl-Thiem-Klinikum, 2. Medizinische Klinik, Thiemstr. 111, 03048 Cottbus, Germany; 6 Charité Comprehensive Cancer Center, Charitéplatz 1, 10117 Berlin, Germany; 7 Helios Klinikum Berlin-Buch, Schwanebecker Chaussee 50, 13125 Berlin, Germany; 8 Charité University Medicine Berlin, Institute of Pathology, Chariteplatz 1, 10117 Berlin, Germany; 9 University Hospital Muenster, Department of Medicine A, Hematology, Oncology and Pneumology, Albert-Schweitzer-Campus 1, 48149 Muenster, Germany; 10 Johannes-Gutenberg University of Mainz, Institute of Molecular Biology, Ackermannweg 4, 55128 Mainz, Germany; 11 Humboldt-University, Department of Biology, Invalidenstr. 43, 10115 Berlin, Germany

Correction to: *Scientific Reports* https://doi.org/10.1038/s41598-018-19201-8, published online 05 February 2018

The Acknowledgements section in this Article is incomplete.

“This work was supported by the Deutsche Krebshilfe e.V., Bonn, Germany, grant 110525 to K.W. and A.L. W.E.B.’s laboratory is supported by Deutsche Forschungsgemeinschaft (DFG), grant EXC 1003. All authors declare that there are no competing financial interests related to this manuscript. The authors thank Wei Chen, Claudia Langnick and Mirjam Feldkamp for deep sequencing services, Berit Pfitzner for help with the MC samples and Romy Leu for excellent technical assistance.”

should read:

“This work was supported by the Deutsche Krebshilfe e.V., Bonn, Germany, grant 110525 to K.W. and A.L. W.E.B.’s laboratory is supported by Deutsche Forschungsgemeinschaft (DFG), grant EXC 1003. All authors declare that there are no competing financial interests related to this manuscript. The authors thank Wei Chen, Claudia Langnick and Mirjam Feldkamp for deep sequencing services, Berit Pfitzner for help with the MC samples and Romy Leu for excellent technical assistance. This work was supported by the fund “Innovative Medical Research” of the University of Münster Medical School (grant WE121504 to K.W).”

