# Supplementary material for: Loss-of-function uORF mutations in human malignancies
Source: Sci Rep. 2018 Feb 5;8:2395. doi: 10.1038/s41598-018-19201-8 (PMC5799362; doi:10.1038/s41598-018-19201-8)
Supplement: Supplementary file 1 — Supplementary Information [file 41598_2018_19201_MOESM1_ESM.pdf]

Supplementary information concerning the manuscript:

Loss-of-function uORF mutations in human malignancies

Julia Schulz[1], Nancy Mah[2], Martin Neuenschwander[3], Tabea Kischka[4], Richard Ratei[5], Peter M. Schlag[6], Esmeralda Castaños-Vélez[6], Iduna Fichtner[1], Per-Ulf Tunn[7], Carsten Denkert[8], Oliver Klaas[9], Wolfgang E. Berdel[9], Jens P. von Kries[3], Wojciech Makalowski[4], Miguel A. Andrade-Navarro[10], Achim Leutz[1,11,*], Klaus Wethmar[1,9,*]

1) Max-Delbrueck-Center for Molecular Medicine, Robert-Roessle-Str. 10, 13125 Berlin, Germany.
2) Charité University Medicine Berlin, Campus Virchow-Klinikum, Berlin-Brandenburg Center for Regenerative Therapies, Augustenburger Platz 1, 13353 Berlin, Germany.
3) Leibniz Institute für Molekulare Pharmakologie, Robert-Roessle-Str. 10, 13125 Berlin, Germany.
4) Institute of Bioinformatics, University of Muenster, Niels-Stensen-Straße 14, 48149 Münster, Germany.
5) Charité University Medicine Berlin, Campus Robert-Roessle, Department of Hematology, Oncology and Cancer Immunology, Lindenberger Weg 80, 13125 Berlin, Germany.
6) Charité Comprehensive Cancer Center, Charitéplatz 1, 10117 Berlin, Germany.
7) Carl-Thiem-Klinikum, 2. Medizinische Klinik, Thiemstr. 111, 03048 Cottbus, Germany.
8) Charité University Medicine Berlin, Chariteplatz 1, 10117 Berlin, Germany.
9) University Hospital Muenster, Department of Medicine A, Hematology, Oncology and Pneumology, Albert-Schweitzer-Campus 1, 48149 Muenster, Germany.
10) Johannes-Gutenberg University of Mainz, Institute of Molecular Biology, Ackermannweg 4, 55128 Mainz, Germany.
11) Humboldt-University, Department of Biology, Invalidenstr. 43, 10115 Berlin, Germany.

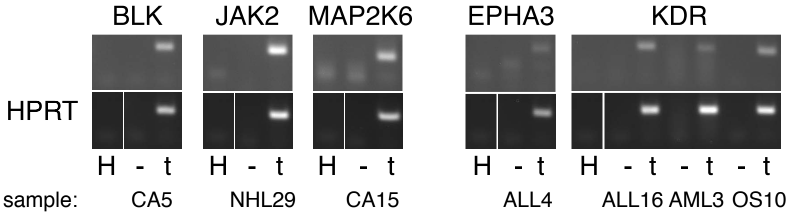

Supplementary Figure 1: Expression of transcripts in affected tumor samples. The expression of indicated transcripts was analyzed by semi-quantitative real-time PCRs following reverse transcriptions of primary RNA isolated from affected cancer samples. Letters below the blots indicate PCR templates: (H) water, (R) non-reverse transcribed RNA, and (D) cDNA.

The transcript of HPRT was analyzed as positive expression control. For each sample, all HPRT PCR products were run on the same gel.

CA – colon adenocarcinoma, NHL – non-Hodgkin lymphoma, ALL – acute lymphoblastic leukemia, AML – acute myeloid leukemia, OS – osteosarcoma.

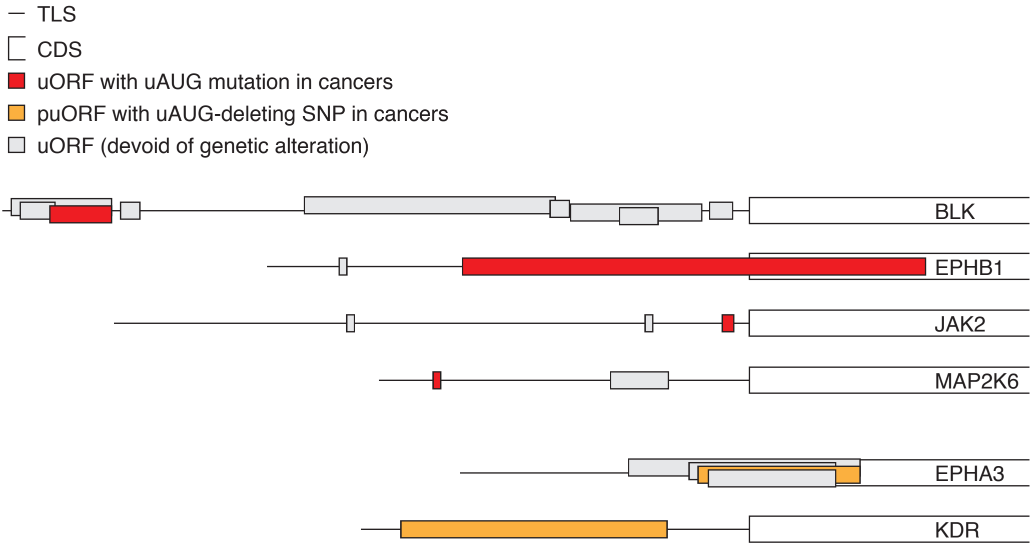

Supplementary Figure 2: Schematic representations of uAUG-mutant or polymorphic TLSs.

Upstream ORFs affected by uAUG-associated mutations and polymorphisms (p) are highlighted by red and orange colors, additional uORFs that remain devoid of genetic alterations in the analyzed set of human cancers are displayed in gray.

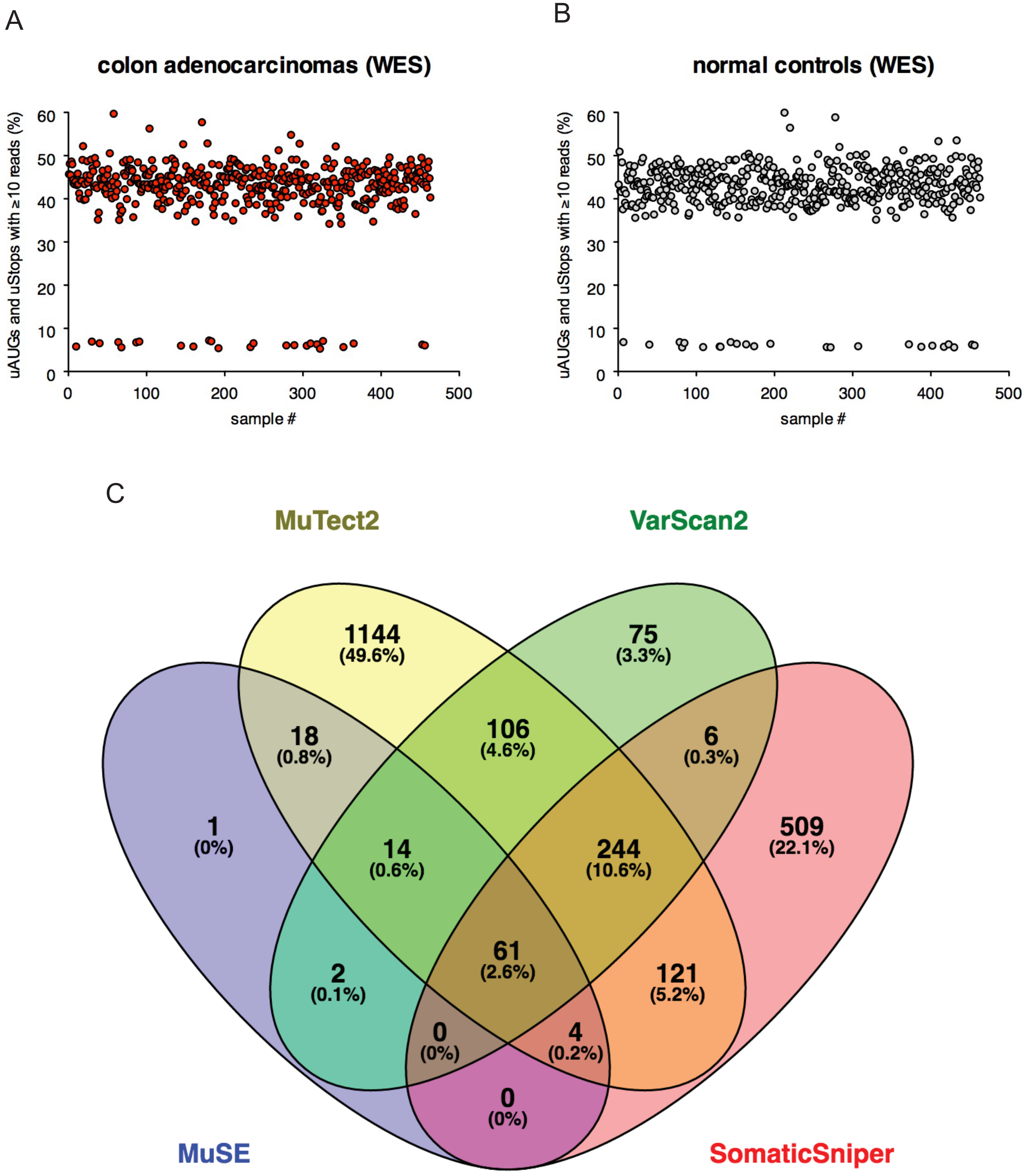

Supplementary Figure 3: uORF-coverage and somatic mutations in colon adenocarcinoma.

(**A and B**) Read coverage at uAUG and uStop codons in whole exome sequencing (WES) datasets of colon adenocarcinoma and corresponding normal controls. WES datasets were obtained trough NCI´s Genomic Data Commons portal (https://gdc-portal.nci.nih.gov).

(**C**) Venn diagram displaying the number of somatic mutations in WES-data of colon adenocarcinoma, as detected by indicated SNP-calling platforms.
